# A Microfluidic Biosensor Based on Magnetic Nanoparticle Separation, Quantum Dots Labeling and MnO_2_ Nanoflower Amplification for Rapid and Sensitive Detection of *Salmonella* Typhimurium

**DOI:** 10.3390/mi11030281

**Published:** 2020-03-09

**Authors:** Li Hao, Li Xue, Fengchun Huang, Gaozhe Cai, Wuzhen Qi, Miao Zhang, Qing’an Han, Zengli Wang, Jianhan Lin

**Affiliations:** 1 Key Laboratory of Agricultural Information Acquisition Technology, Ministry of Agriculture and Rural Affairs, China Agricultural University, Beijing 100083, China; haoli123@cau.edu.cn (L.H.); wuzhen.qi@cau.edu.cn (W.Q.); 2 Key Laboratory of Modern Precision Agriculture System Integration Research, Ministry of Education, China Agricultural University, Beijing 100083, China; li_xue@cau.edu.cn (L.X.); fengchunhuang@cau.edu.cn (F.H.); gaozhe@cau.edu.cn (G.C.); zhangmiao@cau.edu.cn (M.Z.); 3 Veterinary Laboratory, Hebei Animal Disease Control Center, Shijiazhuang 050035, China; hanqingan2004@sina.com (Q.H.); wangzlh891220@163.com (Z.W.)

**Keywords:** Microfluidic biosensor, manganese dioxide nanoflowers, quantum dots, magnetic nanoparticles, *Salmonella* Typhimurium

## Abstract

Screening of foodborne pathogens is an effective way to prevent microbial food poisoning. A microfluidic biosensor was developed for rapid and sensitive detection of *Salmonella* Typhimurium using quantum dots (QDs) as fluorescent probes for sensor readout and manganese dioxide nanoflowers (MnO_2_ NFs) and as QDs nanocarriers for signal amplification. Prior to testing, amino-modified MnO_2_ nanoflowers (MnO_2_-NH_2_ NFs) were conjugated with carboxyl-modified QDs through EDC/NHSS method to form MnO_2_-QD NFs, and MnO_2_-QD NFs were functionalized with polyclonal antibodies (pAbs) to form MnO_2_-QD-pAb NFs. First, the mixture of target *Salmonella* Typhimurium cells and magnetic nanoparticles (MNPs) modified with monoclonal antibodies (mAbs) was injected with MnO_2_-QD-pAb NFs into a microfluidic chip to form MNP-bacteria-QD-MnO_2_ complexes. Then, glutathione (GSH) was injected to dissolve MnO_2_ on the complexes into Mn^2+^, resulting in the release of QDs. Finally, fluorescent intensity of the released QDs was measured using the fluorescent detector to determine the amount of *Salmonella*. A linear relationship between fluorescent intensity and bacterial concentration from 1.0 × 10^2^ to 1.0 × 10^7^ CFU/mL was found with a low detection limit of 43 CFU/mL and mean recovery of 99.7% for *Salmonella* in spiked chicken meats, indicating the feasibility of this biosensor for practical applications.

## 1. Introduction

Food safety has become one of the most important issues in the world. Foodborne pathogenic bacteria are the main cause of foodborne illnesses, including *Escherichia coli* O157:H7, *Listeria monocytogenes*, and *Salmonella* etc. At present, available methods for detecting these bacteria include culture plating, enzyme-linked immunosorbent assay (ELISA), and polymerase chain reaction (PCR), etc. However, they either require a long time, need professional operation, or lack sensitivity. Therefore, simple, fast and sensitive methods for pathogenic bacteria detection are needed to ensure food safety.

In recent years, various biosensors have been reported for determination of foodborne bacteria, such as electrochemical [1,2], quartz crystal microbalance [3,4] and surface plasmon resonance [5,6], etc. They have attracted great attention because of their simple operation, short time, miniaturized size, and high sensitivity. Optical biosensors mainly rely on the measurement of absorbance [7,8], fluorescence [9,10] or Raman scattering [11,12]. Among them, fluorescent biosensors have shown their characteristics of contactless detection, high sensitivity, and inexpensive instrumentation. To date, many fluorescent materials have been employed to develop various fluorescent biosensors, including organic dyes [13,14], up-converting nanoparticles [15,16] and fluorescent microspheres [17,18], etc. As is well known, quantum dots (QDs) are an excellent fluorescent probe with the advantages of wide excitation range, strong fluorescent signal and long fluorescent lifetime, and are often used for fluorescent biosensing [19,20,21].

To make better use of QDs, they are often combined with other functional nanomaterials, such as Graphene and TiO_2_, to improve the sensitivity [22,23]. As an easy-to-synthesize and non-toxic nanomaterial with high specific surface area, manganese dioxide nanoflowers (MnO_2_ NFs) have been used to develop biosensors for detection of different targets, such as Tumor cells, GSH and glucose [24,25,26]. Besides, MnO_2_ can be easily reduced to Mn^2+^ using glutathione (GSH) [27], H_2_O_2_ [28], thiocholine [29], etc., resulting in decomposition of MnO_2_ NFs.

In the past decades, microfluidic chips have drawn numerous attentions in biochemical analysis field due to high integration, complete automation and small size [30,31], and have been applied for detecting tumor cells [32], proteins [33] and pathogens [34,35,36,37], etc. An interesting study was a microfluidic chip containing a passive micromixer with triangular baffles and circular obstructions, which was demonstrated with a high mixing index of 0.99 under optimal conditions [38]. Therefore, the combination of QDs labeling, MnO_2_ decomposition and microfluidic chip might be a promising method to detect foodborne pathogens with higher sensitivity, easier operation and shorter time.

**Scheme 1:**
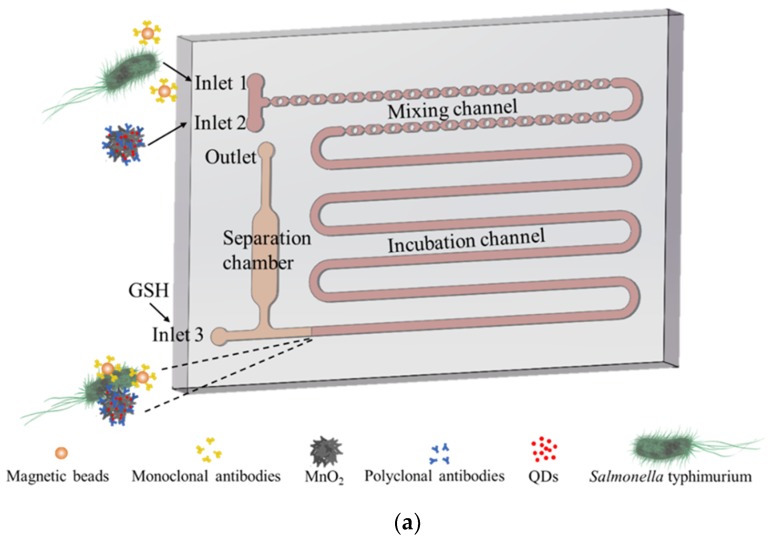

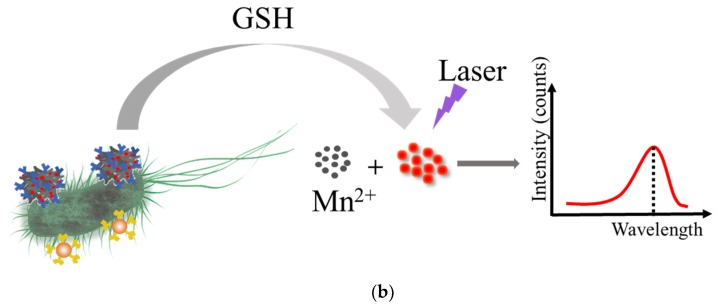
(**a**) The schematic of the microfluidic chip; (**b**) The principle of the fluorescent biosensor for rapid and sensitive detection of *Salmonella* Typhimurium.

In this study, we developed an optical biosensor using QDs for fluorescent labeling, MnO_2_ NFs for signal amplification, and microfluidic chip for automatic operation to rapidly and sensitively determine *Salmonella* Typhimurium. As shown in Scheme 1, the bacterial sample was first injected with MnO_2_-QDs-pAb NFs and magnetic nanoparticles (MNPs) modified with monoclonal antibody against *Salmonella* Typhimurium into the microfluidic chip. Then, the MNP-bacteria-QD-MnO_2_ complexes were formed after efficient mixing and sufficient incubation. After the complexes were captured in the separation chamber using the external magnetic field, GSH was finally injected into the chamber to decompose the MnO_2_ NFs into Mn^2+^ for releasing the QDs, whose fluorescent intensity was measured by the optical detector to determine the amount of the *Salmonella* cells.

## 2. Materials and Methods

### 2.1. Materials

Phosphate buffered saline (PBS, 10 mM, pH 7.4) from Sigma Aldrich (St. Louis, MO, USA) was used as a buffer solution. KMnO_4_ from Sinopharm chemical (Beijing, China), and HCl and polyvinylpyrrolidone (PVP) from Sigma Aldrich (St. Louis, MO, USA) were used to synthesize MnO_2_ NFs. (3-aminopropyl) triethoxysilane (APTES) from Aladdin (Shanghai, China) was used to modify MnO_2_ NFs with amino groups. The carboxylated CdSe/ZnS core/shell QDs (emission wavelength: 651 nm, concentration: 1 µM) from Mesolight (Suzhou, China) were used as fluorescent probes. The monoclonal antibodies against *Salmonella* (mAbs, 1 mg/mL) from Meridian (Memphis, TN, USA) and the polyclonal antibodies against *Salmonella* (pAbs, 2.5 mg/mL) from Fitzgerald (Acton, MA, USA) were used to specifically recognize the *Salmonella* Typhimurium cells. N-(3-Dimethylaminopropyl)-N’-ethylcarbodiimide hydrochloride (EDC·HCl) and N-Hydroxysulfosuccinimide sodium (NHSS) from Sigma Aldrich (St. Louis, MO, USA) were used to prepare theMnO_2_-QD-pAb NFs. The biotin labeling kit from Elabscience (Wuhan, China) was used to biotinylate the mAbs. The streptavidin-modified MNPs with diameters of 150 nm from Ocean Nanotech (MHS-150-10, San Diego, CA, USA) were used to magnetically separate the *Salmonella* cells. Bovine serum albumin (BSA) from Coolaber (Beijing, China) was used for blocking. Luria-Bertani (LB) medium from Aoboxing Biotech (Beijing, China) was used for bacterial culture. The silicone elastomer kit (Sylgard 184) from Dow Corning (Midland, MI, USA) was used to fabricate the poly (dimethoxy) silane (PDMS) channel. The Objet24 3D printer from Stratasys (Eden Prairie, MN, USA) was used to fabricate the mold of the channel. The NdFeB magnets (grade: N52) bought locally were used to generate the external magnetic field. Deionized water produced by Advantage A10 from Millipore (18.2 MΩ∙cm, Billerica, MA, USA) was used to prepare all the solutions.

### 2.2. Design and Fabrication of the Microfluidic Chip

The design of the microfluidic chip with the convergence-divergence mixing channel was inspired by a previous report [37]. The microfluidic chip mainly consisted of three parts: (1) the mixing channel with the structure of convergence-divergence with a width of 1 mm and height of 400 µm for mixing the bacterial sample with the MNPs and the MnO_2_-QD-pAb NFs; (2) the incubating channel with the same width and height for forming the MNP-bacteria-QD-MnO_2_ complexes; and (3) the separation chamber with a length of 11 mm, width of 3 mm and height of 1 mm for capturing the MNP-bacteria-QD-MnO_2_ complexes with the external magnetic field and reducing MnO_2_ NFs into Mn^2+^ with GSH.

The microfluidic chip was fabricated based on 3D printing and surface plasma bonding. As shown in Scheme 1b, the 3D mold was drawn in stl format using the Solidworks software (Dassault Systèmes SolidWorks Corporation, Waltham, MA, USA) and printed by the Objet24 3D printer. Prior to use, the mold was thoroughly washed by deionized water to remove residual supporting materials. The prepolymer of PDMS and the curing agent were mixed at the ratio of 10:1 and poured into the mold, followed by curing at 65 °C for 12 h after degassing for 15 min in vacuum to remove bubbles. The PDMS replica was peeled off and bonded onto the glass slide to form the microfluidic chip after surface plasmon treatment (Harrick Plasma, Ithaca, NY, USA) and baked at 65 °C for aging to finally obtain the microfluidic chip.

### 2.3. Synthesis of the MnO_2_-QD-pAb NFs

The MnO_2_-QD-pAb NFs were synthesized based on our previous study [39]. First, 10 mg of MnO_2_ NFs were centrifuged at 10,000 rpm for 10 min to remove the supernatant and resuspended with 2 mL of ethanol. Then, 1 mL of APTES was added and incubated at 37 °C with gentle stirring for 12 h to obtain NH_2_-MnO_2_ NFs in deionized water. After 5 µL of QDs (1 µM), 48 µL of EDC (1 mg/mL) and 11 µL of NHSS (1 mg/mL) were transferred into a centrifuge tube containing 1 mL of PB (pH 6.0, 0.01 M) and incubated for 1 h, 700 µL of NH_2_-MnO_2_ NFs (1 mg/mL) were added and incubated for 2 h at room temperature, followed by centrifugation to obtain the MnO_2_-QD NFs. Finally, 14 µL of polyclonal antibodies (2.5 mg/mL) and 500 µL of MnO_2_-QD NFs (1 mg/mL) were incubated with 10 µg of EDC (1 mg/mL) for 30 min, and 210 µL of 10% BSA (w/v) and 30 µL of EDC were added and incubated for another 1 h, followed by centrifugation and resuspending in 500 µL of PB containing 1% BSA to obtain the MnO_2_-QD-pAb NFs, which were stored at 4 °C for further use.

### 2.4. Detection of the Target Bacteria in Pure Cultures

The detection of the target bacteria was based on the forming of the MNP-bacteria-QD-MnO_2_ sandwich complexes and the release of the QDs from the MnO_2_ NFs on the complexes. First, 1 mL of the bacterial culture containing *Salmonella* Typhimurium at each concentration of 1.0 × 10^2^–1.0 × 10^7^ CFU/mL was mixed with 20 µg of MNPs modified with monoclonal antibody against *Salmonella* Typhimurium and 50 µg of the MnO_2_-QD-pAb NFs, and injected into the microfluidic chip through inlet 1 (bacterial culture and MNPs) and inlet 2 (MnO_2_-QD-pAb NFs) at the flow rate of 25 µL/min using two precise syringe pumps (Pump 11 elite, Harvard Apparatus, Holliston, MA, USA), respectively. The MNP-bacteria-QD-MnO_2_ complexes were formed and captured in the separation chamber by applying the magnetic field generated by two repelling NdFeB magnets at the bottom of the chamber. Then, 500 µL of PBST (PBS containing 0.05% Tween 20) was injected into the chamber to remove the residual background and excessive MnO_2_-QD-pAb NFs. After 100 µL of GSH (20 mM) was injected to the chamber through inlet 3 and incubated for 15 min to release the QDs from the MnO_2_ NFs, the released QDs were finally flushed out and measured by the optical detector to determine the amount of target bacteria. The portable optical detector from Ocean Optics (Dunedin, FL, USA) consisted of a USB4000-UV-VIS spectrophotometer with a spectral range of 200–850 nm for fluorescence detection, a USB-LS-450 blue LED pulsed light source for fluorescence excitation, a QR400-7 UV-Vis optical probe for optical transmission, and a Spectra-Suite software for data analysis.

### 2.5. Detection of the Target Bacteria in Spiked Samples

The chicken samples were purchased from a local supermarket. First, 25 g of chicken sample was added into 225 mL of sterile PBS and homogenized (BagMixer, Interscience, Mourjou, France) for 4 min, followed by standing for 5 min to obtain the supernatant. Then, different concentrations of the Salmonella Typhimurium cells were added into the supernatant to obtain the spiked chicken samples with bacterial concentrations ranging from 1.0 × 10^2^ to 1.0 × 10^7^ CFU/mL. Finally, the spiked chicken samples were detected using this biosensor.

## 3. Results

### 3.1. Simulation of the Microfluidic Chip

**Figure 1:**
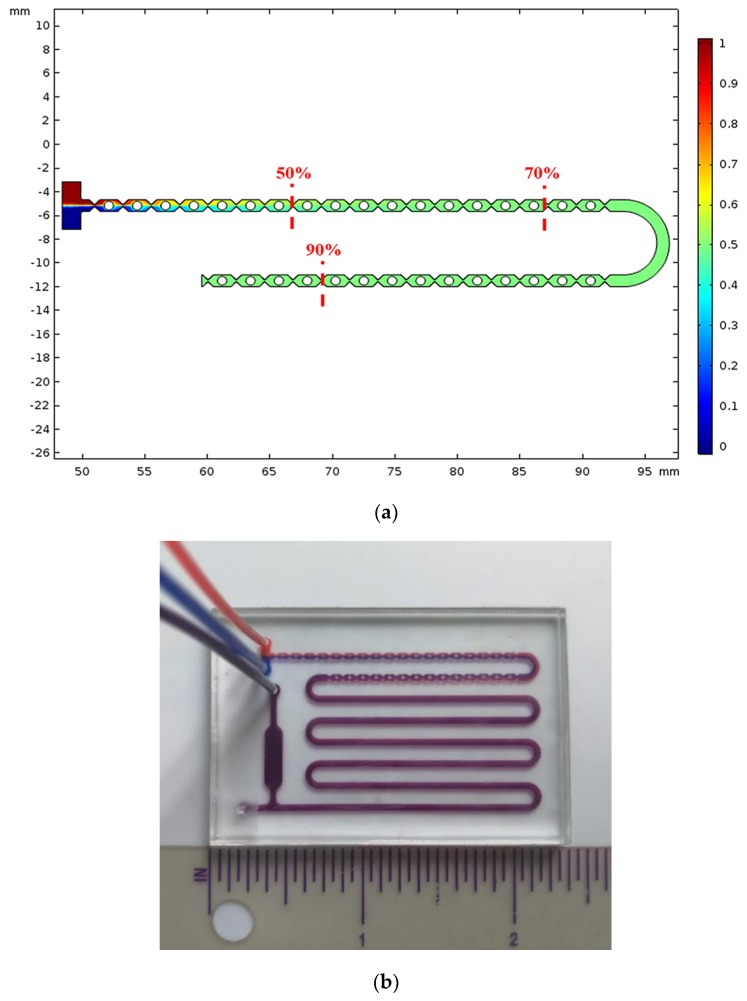
(**a**) The simulation on mixing efficiency of the mixing channel; (**b**) The mixing of two inks in the microfluidic chip.

The micromixer is the key to the microfluidic chip, which has a great impact on the detection time and sensitivity of this biosensor. COMSOL software (COMSOL Inc., Stockholm, Sweden) was used to simulate the mixer with a convergence-divergence structure and evaluate its mixing efficiency. As shown in Figure 1a, the mixing efficiency reaches 50%, 70% and 90%, when the number of the convergence-divergence structures is 7, 16 and 28, respectively. This indicates that the convergence-divergence mixer has an excellent mixing efficiency. To ensure complete mixing of MNPs modified with monoclonal antibody against Salmonella Typhimurium, the MnO_2_-QD-pAb NFs, and the target bacteria, 32 convergence-divergence structures and a serpentine incubating channel were used. As shown in Figure 1b, the microfluidic chip has a length of ~6 cm and width of ~4 cm. To further verify the mixing efficiency of the chip, red and blue inks were injected into the chip at the same rate of 25 µL/min. As can be seen from Figure 1b, blue and red inks were fully mixed after flowing through the mixing channel.

### 3.2. Characterization of the MnO_2_ NFs and MnO_2_-QD NFs

**Figure 2:**
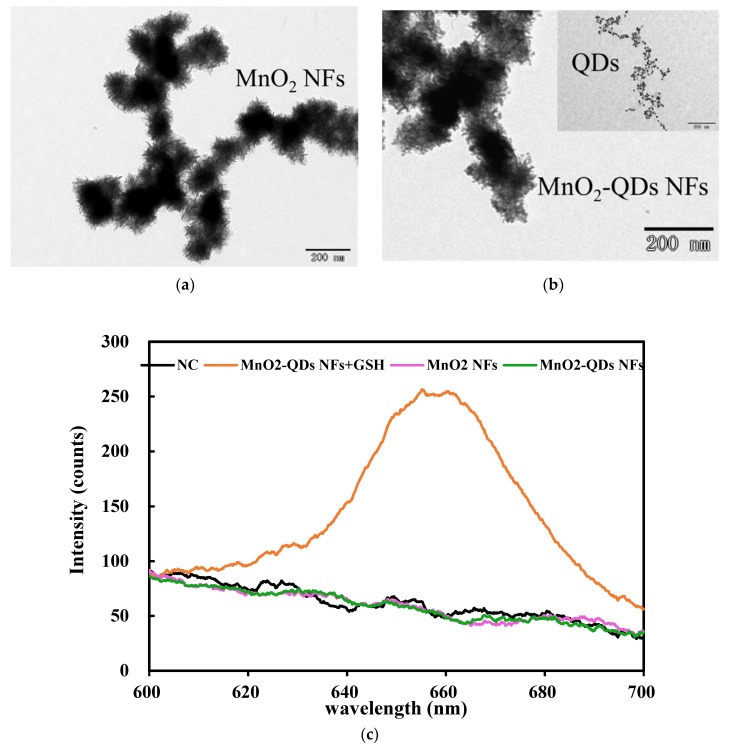
(**a**) The TEM image of the MnO_2_ NFs; (**b**) The TEM image of the MnO_2_-QD NFs. The inset is the TEM image of the QDs; (**c**) The fluorescent spectra of the QDs, MnO_2_ NFs, MnO_2_-QD NFs, and the mixture of the MnO_2_-QD NFs and GSH.

The MnO_2_ NFs and their derivates are the key materials of this biosensor. Thus, transmission electron microscopy (TEM) was used to characterize the MnO_2_ NFs and MnO_2_-QD NFs. As shown in Figure 2a, the MnO_2_ NFs have many flakes deriving from their backbones, and their average size is ~200 nm. As shown in Figure 2b, there are many small dots with an average size of ~10 nm on the MnO_2_ NFs, which is consistent with the size of QDs (see the insert of Figure 2b), indicating successful synthesis of the MnO_2_-QD NFs. Besides, to verify the basic concept of this biosensor, the fluorescent intensities of the MnO_2_ NFs, the MnO_2_-QD NFs and the mixture of MnO_2_-QD NFs and GSH were measured using the optical detector. Figure 2c shows that only the mixture of MnO_2_-QD NFs and GSH has a fluorescent signal due to the decomposition of the MnO_2_ NFs, resulting in the release of the QDs. The MnO_2_ NFs do not have fluorescent signals, indicating that there are only little background noises from the MnO_2_ NFs. The MnO_2_-QD NFs also do not have fluorescent signals since the fluorescence of the QDs is absorbed and/or blocked by MnO_2_. To further investigate the release of the QDs from the MnO_2_-QD NFs, twofold dilutions of the MnO_2_-QD NFs were prepared and incubated with 100 µL of 20 mM GSH for 15 min. The fluorescent intensity of the released QDs was detected and shown in Figure S1. It shows that there is a good linear relationship between the fluorescent intensity of the released QDs and the concentration of the MnO_2_-QD NFs.

### 3.3. Optimization of the Microfluidic Biosensor

The amount of QDs, the amount of MnO_2_-QD-pAb NFs, and the flow rate have a great impact on the sensitivity of this microfluidic biosensor. The detailed procedures for their optimization can be found in the Supplementary Materials. As shown in Figure S2, the optimal amount of 50 pmol for the QDs, the optimal amount of 50 µg for the MnO_2_-QD-pAb NFs, and the optimal flow rate of 25 µL/min could be obtained and were used in this study.

### 3.4. Simulation of the Magnetic Field

**Figure 3:**
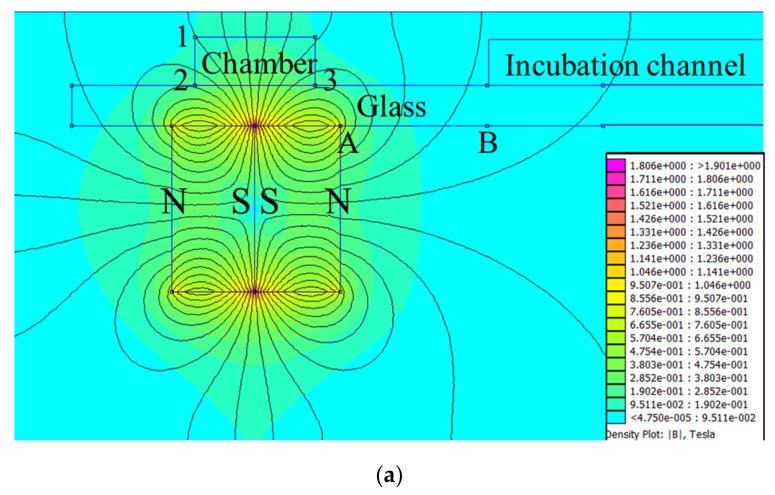

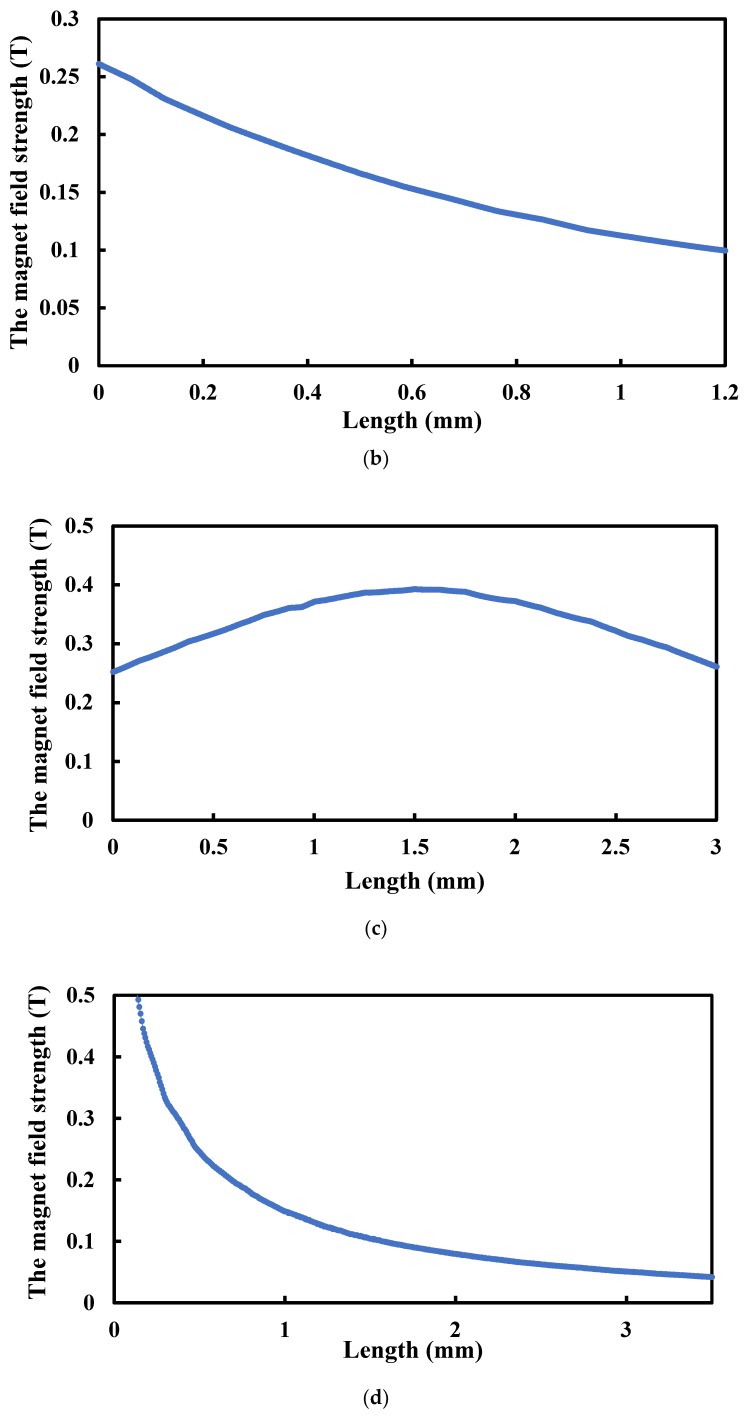
(**a**) The distribution of the magnetic field; (**b**) The strength of the magnetic field in the separation chamber at the vertical direction; (**c**) The strength of the magnetic field in the separation chamber at the horizontal direction; (**d**) The strength of the magnetic field from the separation chamber to the incubation channel.

The magnetic field for capturing the MNP-bacteria-QD-MnO_2_ complexes in the separation chamber is important in the development of this biosensor. To better investigate the distribution of the magnetic field in the microfluidic chip, the Finite Element Method Magnetics (FEMM) Software (Developed by David Meeker, and available from the website: http://www.femm.info/) was used to simulate the magnetic field generated by two repelling magnets (material: NdFeB, grade: N52, dimension: 9 × 4 × 2 mm). Figure 3a shows the distribution of the magnetic field at the chip. The strength of the magnetic field in the separation chamber changes from 0.10 T to 0.26 T at the vertical horizontal direction (Point 2 to 1, Figure 3b) and from 0.25 to 0.39 T at the horizontal direction (Point 2 to 3, Figure 3c), which was strong enough to capture the MNPs with the size of 150 nm. Besides, to check if the magnetic field has an impact on the MNPs and their conjugates when they flowed through the incubation channel, the strength of the magnetic field at the horizontal direction from the right magnet was obtained from FEMM simulation. As shown in Figure 3d, the strength of the magnetic field at the closest position (point B) of the incubation channel is only 0.05 T, which is not strong enough to capture the flowing MNPs or their conjugates [40]. To further verify this, 20 µg of the MNPs in 1 mL of PBS were injected into the chip with the flow rate of 25 µL/min. As shown in Figure S3, when the magnetic field is applied, almost all MNPs are captured in the separation chamber other than in the incubation channel.

### 3.5. Calibration Model of This Biosensor

**Figure 4:**
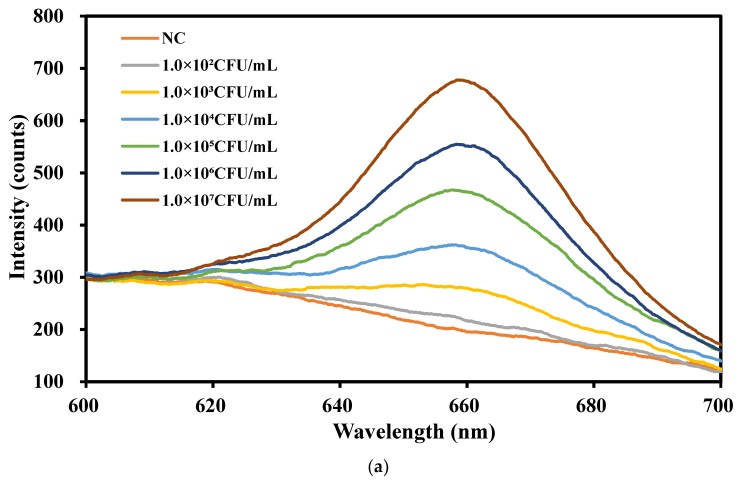

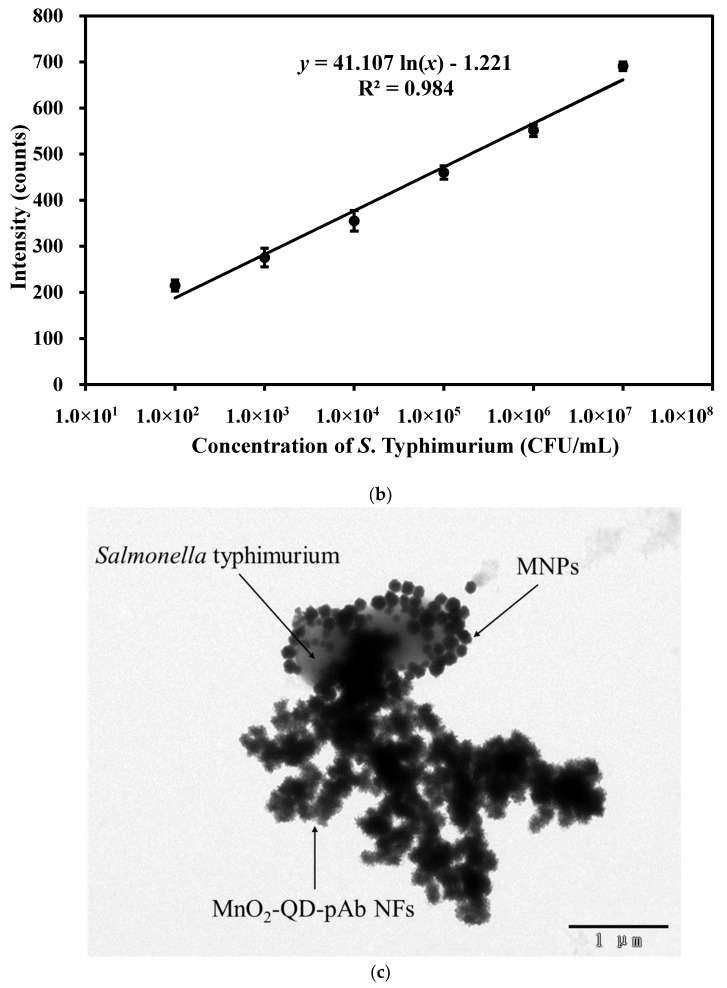
(**a**) The spectra of the MNP-bacteria-MnO_2_-QD complexes at different concentrations from 1.0 × 10^2^ CFU/mL to 1.0 × 10^7^ CFU/mL; (**b**) The linear relationship between the fluorescent intensity and the bacterial concentration; (**c**) The TEM image of the MNP-bacteria-MnO_2_-QD complexes.

The calibration model of this biosensor is the basis for quantitative determination of unknown concentrations of target bacteria. Different concentrations of the Salmonella Typhimurium cells ranging from 1.0 × 10^2^ CFU/mL to 1.0 × 10^7^ CFU/mL were detected using this biosensor under optimal conditions. As shown in Figure 4a, the fluorescent intensity at the characteristic wavelength of 661 nm increases from about 200 counts to about 700 counts when the bacterial concentration changes from 10^2^ CFU/mL to 10^7^ CFU/mL. As shown in Figure 4b, a good linear relationship between the fluorescent intensity (*I*) at 661 nm and the bacterial concentration (*C*) is found and can be expressed as *I* = 41.107 ln(*C*) − 1.221 (R^2^ = 0.984). Based on three times the signal-to-noise ratio, the low detection limit of this biosensor was calculated to be 43 CFU/mL. To further confirm the formation of the MNP-bacteria-MnO_2_-QD complexes, transmission electron microscope was conducted and the image is shown in Figure 4c, indicating successful formation of the complexes. The high sensitivity of this biosensor could be attributed to these reasons: (1) the high load of the MnO_2_ NFs with the QDs, resulting in stronger fluorescent signals; (2) the effective release of the QDs by GSH into aqueous solution, resulting in much less absorption of the fluorescence by the MnO_2_ NFs and no blocking of the fluorescence; (3) the good control of the background noise by automatic operations and continuous-flow washing in the microfluidic chip, resulting in less background noise; (4) the efficient concentration of the MNP-bacteria-MnO_2_-QD complexes in the separation chamber by the magnetic field, resulting in stronger fluorescent signals.

### 3.6. Applicability of This Biosensor

**Figure 5:**
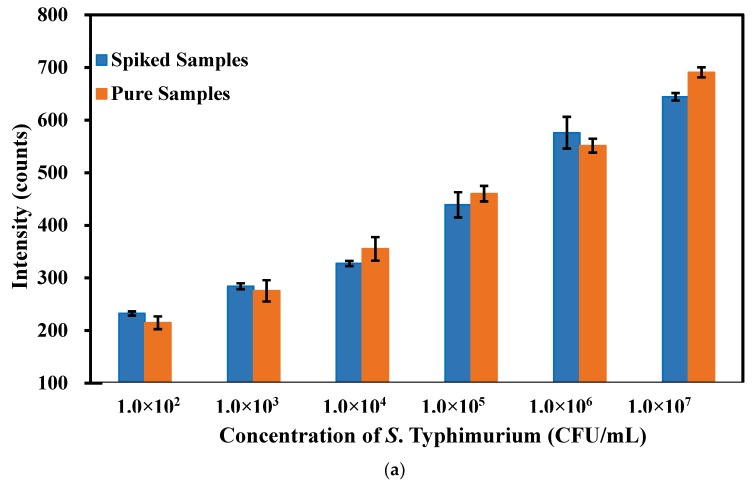

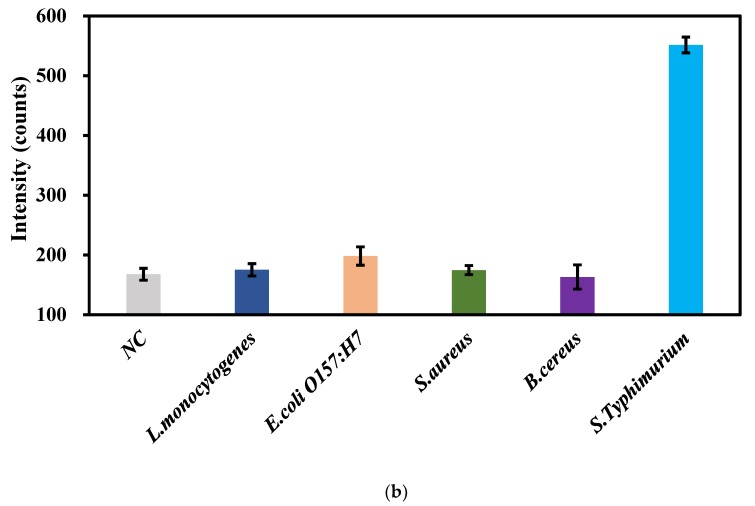
(**a**) Detection of chicken samples spiked with *Salmonella* Typhimurium (N = 3); (**b**) Detection of *L. monocytogenes*, *E. coli* O157:H7, *S. aureus*, *B. cereus*, and *S*. Typhimurium at the same concentration of 10^5^ CFU/mL, and the negative control (N = 3).

To evaluate the applicability of this biosensor for detection of *Salmonella* Typhimurium in food samples, chicken meats were purchased from the local supermarket and used as a real sample model. Prior to testing, 25 g of a chicken meat was added into 225 mL of PBS to obtain the supernatant. Different concentrations of target *Salmonella* Typhimurium cells were added into the supernatant to prepare the spiked chicken samples with the bacterial concentrations ranging from 1.0 × 10^2^ to 1.0 × 10^7^ CFU/mL, which were detected by this biosensor. As shown in Figure 5a, there are only slight differences on the fluorescent intensity between the spiked chicken sample and the pure culture at each concentration, which are mainly caused by non-specific binding of the impurities in the spiked samples. The recoveries (the ratio of the readout of the biosensor to the original concentration) for the concentrations from 1.0 × 10^2^ to 1.0 × 10^7^ CFU/mL were 111.5%, 104.0%, 90.6%, 94.7%, 105.0%, and 92.6%, respectively, and the mean recovery was 99.7%, verifying the applicability of this fluorescent biosensor for detection of *Salmonella* Typhimurium in real samples.

The specificity of this biosensor was evaluated using *L. monocytogenes*, *E. coli* O157:H7, *S. aureus* and *B. cereus* as non-target bacteria. The negative control (PBS) and the same concentration (1.0 × 10^5^ CFU/mL) of the target and non-target bacteria were detected using this biosensor. As shown in Figure 5b, it was obvious that the fluorescent intensity of the target bacteria is much higher than those of the non-target bacteria, which are close to that of the negative control, indicating that this biosensor has a good specificity. This could be attributed to the good specificity of the monoclonal and polyclonal antibodies against the target bacteria.

## 4. Conclusions

In summary, a microfluidic biosensor using MNPs for separation and enrichment, QDs for fluorescent labeling, MnO_2_ NFs for signal amplification, and GSH for QDs release was successfully developed for rapid and sensitive detection of *Salmonella* Typhimurium. Under the optimal conditions, this biosensor had a wide linear detection range from 1.0 × 10^2^ to 1.0 × 10^7^ CFU/mL and was able to detect *Salmonella* Typhimurium as low as 43 CFU/mL in spiked chicken meats. The MnO_2_ NFs were demonstrated to have high specific surface area to load more QDs for signal amplification to improve the sensitivity of the biosensor. More importantly, this biosensor integrated the mixing, immune reaction, magnetic separation, and QDs release onto one single microfluidic chip, which was promising for in-field applications in foodborne pathogen detection. This biosensor also has the potential to be extended for the detection of other foodborne pathogens by changing the biological recognition elements.

## Supplementary Materials

The following are available online at https://www.mdpi.com/2072-666X/11/3/281/s1, Figure S1: The fluorescence of the released QDs on 2-fold dilutions of the MnO_2_-QD NFs, Figure S2: (a) Optimization of the amount of the QDs; (b) Optimization of the amount of the MnO_2_-QD-pAb NFs; (c) Optimization of the flow rate, Figure S3: (a) Difference in separation chamber with or without magnets; (b) The color of supernatant of MNPs, suspended MNPs after separating and original MNPs. Procedure 1: Preparation of the bacteria, Procedure 2: Preparation of the MnO_2_ NFs.
